# Sodium Hypochlorite Irrigation and Its Effect on Bond Strength to Dentin

**DOI:** 10.1155/2017/1930360

**Published:** 2017-08-20

**Authors:** Tariq S. Abuhaimed, Ensanya A. Abou Neel

**Affiliations:** ^1^Division of Biomaterials, Operative Dentistry Department, King Abdulaziz University, Jeddah, Saudi Arabia; ^2^Biomaterials Department, Faculty of Dentistry, Tanta University, Tanta, Egypt; ^3^Biomaterials and Tissue Engineering Division, Eastman Dental Institute, UCL, 256 Gray's Inn Road, London WC1X 8LD, UK

## Abstract

Effective shaping and cleaning of root canals are essential for the success of endodontic treatment. Due to the complex anatomy of root canal spaces, the use of various instrumentation techniques alone is not effective in producing bacteria-free root canal spaces. Irrigation, disinfectants, rinses, and intervisit medications are used in conjunction with the mechanical instrumentation to ensure the success of endodontic treatment. Sodium hypochlorite (NaOCl), a halogenated compound, is routinely used to irrigate the root canal during endodontic treatments. NaOCl has been known for its antibacterial action, proteolytic and dissolution capacity, and debridement properties. NaOCl, however, can alter the composition of dentin and hence its interaction with the adhesive resins used to bond the restorative materials to treated dentin. This review therefore covers in depth the action of NaOCl on dentin-adhesive resin bond strength including both enhancement and reduction, then mechanisms proposed for such action, and finally how the adverse action of NaOCl on dentin can be reversed.

## 1. Introduction

Effective shaping and cleaning of root canals are essential for the success of endodontic treatments. Due to the complex anatomy of root canal spaces, the use of various instrumentation techniques alone is not effective in producing bacteria-free root canal spaces [1, 2]. Large areas of root canal dentin could remain untouched by the instruments; this emphasizes the importance of chemical means of cleaning and disinfecting the root canal system [2]. Various chemicals, for example, irrigations, disinfectants, rinses, and intervisit medications are used in conjunction with the mechanical instrumentation to ensure the success of endodontic treatments [1, 3]. Irrigation flushes away all loose, necrotic, or contaminated tissues and materials from the root canal before being pushed into the apical tissues. It also provides lubrication, debridement, destruction of microbes, and dissolution of tissues [4].

Sodium hypochlorite (NaOCl), a halogenated compound, is routinely used to irrigate the root canal during endodontic treatments. It has antimicrobial action [5–8]; in water, sodium hypochlorite ionizes to Na and OCl. Between pH values 4 and 7, chlorine ion exists as hypochlorous acid (HClO) while, at pH above 9, OCl predominates. HClO has a strong antibacterial action compared to OCl due to its ability to disrupt the oxidative phosphorylation and other membrane-associated activities [9]. HClO also exerts a rapid inhibitory effect on mitochondrial function and DNA synthesis of bacteria [10]. In addition to its antibacterial action, sodium hypochlorite has the ability to dissolve the pulpal remnants [11, 12] and organic component of dentin (i.e., nonspecific proteolytic action [3]). It also has the ability to partially neutralize the necrotic tissues or any antigenic or microbial component left in the root canal space [13] and remove all pulpal remnants and predentin on the uninstrumented surfaces [14]. The tissue dissolution capacity and debridement properties can be significantly improved by increasing the temperature and concentration of sodium hypochlorite [15, 16]. The penetration ability to the uninstrumented area of root canal systems can be increased by lowering the surface tension of NaOCl [17]. Regardless of its significant effect on the organic component of dentin, NaOCl has no effect on dentin's inorganic part [18].

Chemicals used during the preparation of root canals, however, can alter the composition and hence the interaction of dentin surface with the restorative materials. In literatures, there are conflicting results on how the treatment of dentin with NaOCl affects the subsequent bond strength. Such variation in response to sodium hypochlorite could be related to the variation in methodology, form (gel versus liquid) and concentration of sodium hypochlorite, time of application [19], and many other factors that will be discussed in detail in this review.

## 2. Effect of Sodium Hypochlorite on Dentin Bonding

Wakabayashi et al. observed that the treatment of dentin with 10% NaOCl after etching with 40% phosphoric acid enhanced the tensile strength of adhesive to dentin. Even after thermocycling (10,000 cycles at 4–60°C), the bond strength was 1.5 times higher than that recorded for etched dentin [20]. Vagas et al. observed that a 2-minute exposure of dentin to 5% NaOCl following acid etching improved the shear bond strength for All-Bond 2 but had no significant effect on Scotchbond Multi-Purpose [21]. Prati et al. observed that treatment of etched (35% phosphoric acid for 20 s) dentin with NaOCl (1.5% for 2 min or 10% for 120 hr) improved the shear bond strength of Optibond FL when compared to etched dentin. The bond strength of Scotchbond MP and 3 M Single Bond to etched/NaOCl-treated dentin was significantly reduced when compared to etched dentin. The bond strength of Prime & Bond 2.0, however, remained unmodified compared to etched dentin. Long term (120 hr) NaOCl treatment of etched dentin produced an exceptional layer of resin-infiltrated mineralized dentin “reverse hybrid layer”; this will be discussed later in this review [22]. Ari et al. observed that the microtensile bond strength to 5% NaOCl-treated dentin has been significantly improved with C&B Metabond compared to with Panavia F and Variolink II [23].

Stevens observed that the pretreatment of dentin with 6% NaOCl (20 min exposure) adversely affects the bond strength of self-etching, self-adhesive resin cements (Speed CEM and Clearfil SA Cement) but not total-etch (Variolink II) adhesives and those with a separate bonding agent (Multilink and Clearfil Esthetic Cement EX) [24]. Similar findings were also observed by Ishizuka et al., who observed that the bond strength of self-etching primer systems (Clearfil Mega Bond) significantly decreased after being applied to 6% NaOCl-treated dentin (1 or 5 or 10 min exposure) while that of total-etch system (Single Bond) did not change [25]. Contradicting results were observed in other studies. Frankenberger et al. observed a significant reduction in dentin push-out bond strength with both total-etch (Scotchbond Multi-Purpose Plus, EBS, and Solid Bond) and self-etch (Prime & Bond 2.1, Syntac Sprint) adhesives with dentin [26]. Nikaido et al. observed a significant reduction in bond strength only with total-etch (Single Bond II) and Superbond C&B [a self-cure resin cement based on 4-methacryloxyethyl trimellitate anhydride in methyl methacrylate initiated by tri-n-butyl borane (4-META/MMA-TBB)], but the bond strength of self-etching primer system (Clearfil Mega Bond) did not significantly change when applied to 5% NaOCl-treated dentin (60 s exposure) [27]. Vongphan et al. observed a significant reduction in bond strength of total-etch system (Single Bond) when bonded to 5.25% NaOCl-treated dentin (10 min exposure) [28]. Perdigão et al. observed that the shear bond strength of two total-etch adhesives (Prime & Bond NT and Single Bond) has been significantly reduced with the application of 10% NaOCl gel on dentin [29]. This reduction was significantly increased with increasing the time of gel application [29]. Morris et al. observed a significant reduction in the tensile bond strength of Metabond resin cements to 5% NaOCl-treated dentin (15–20 min exposure) [30]. Soeno et al. observed a significant reduction in bond strength of a carboxylic (4-META/MMA-TBB) resin to 10 wt% NaOCl-treated dentin (30 s exposure) [31]. A summary of these studies is presented in Table 1.

As observed* the effect of sodium hypochlorite on dentin bonding varies with chemistries of the bonding systems*[32]. Such variation in bonding systems' chemistries was related to the ability of their etchants to remove the degenerated dentin, produced by acid etching, as well as the residual NaOCl, that would interfere with the free radical polymerization reaction of resin cement, from treated dentin [25]. As suggested by some authors, single bond etchants, for example, have the ability to remove the degenerated dentin and residual NaOCl from treated dentin but not the self-etching primer of Mega Bond. According to this suggestion, the bond strength with NaOCl-treated dentin has been improved with single bond adhesives compared to self-etch systems [25]. This assumption can only explain the enhancement but not the reduction of bond strength seen with single bond adhesives. Therefore other factors could play a key part in bond strength to NaOCl-treated dentin, for instance,* the form (solution or gel), concentration, and time of application of sodium hypochlorite [19].* Regardless of the variation in bonding systems and testing methods used, like 6% sodium hypochlorite [24], 5% (for 15–20 min) produced a significant reduction in the tensile resin-dentin bond strength of Metabond resin cements as discussed above [30]. However, when the time of NaOCl application has been reduced, in another study, to 60 s, still a significant reduction in bond strength was achieved with 5% NaOCl [33]. Also 2.5% NaOCl applied for 60 s significantly reduced the pull-out bond strength of dentin to self-adhesive resin cements [34]. On the other hand, using 1% NaOCl (5 ml applied every 5 min for 1 hr) significantly improved the tensile bond strength of dentin [35]. In these studies [30, 33–35], however as highlighted above, the concentration and time of NaOCl application were not the only variables to be considered responsible for the action of NaOCl. *Therefore, the source of dentin samples (animal or human), as well as the age of human or animal from which the samples were collected,* is another factor to be considered.* The storage condition of dentin samples, the regional variation* (coronal versus root dentin/cervical versus middle versus apical root canal dentin) [30],* and location* (superficial versus root canal wall)* of dentin* [25, 36] would also play a part. Variation in* technique used to prepare samples and to measure the bond strength* (shear, tensile, push-out or pull-out, etc.) could be another factor.

Regardless of the wide variation in results, only few studies reported an enhancement in resin bond strength after dentin treatment with sodium hypochlorite. The proposed mechanisms for either enhancement or reduction in dentin bond strength will be discussed in the next section.

## 3. Suggested Mechanisms for the Action of Sodium Hypochlorite on Dentin Bond Strength

### 3.1. Bond Strength Enhancement

Regarding the action of sodium hypochlorite itself, the enhancement in dentin bond strength observed after NaOCl treatment has been related to its deproteinizing action. NaOCl has the ability to dissolve and remove the exposed dentinal collagen, produced by acid etching, and provide a fresh mineralized dentin surface to which the adhesive resin can be applied. This allows a direct adhesion between the adhesive resin and dentin without the resin-reinforced collagen layer, called hybrid layer [20, 21]. According to Prati et al., NaOCl produced an unusual new mechanism of micromechanical retention of resin into mineralized dentin, called “reverse hybrid layer formation” [22]. For the formation of conventional hybrid layer, the technique relies on the use of acid etching to remove the smear layer as well as the mineral phase of dentin (i.e., exposing collagen fibrils). Upon subsequent use of the adhesive resin, the resin infiltrates around the exposed collagen fibrils and replaces the lost mineral phase (i.e., creating a layer of “resin-infiltrated collagen”). The application of NaOCl after acid etching and before the adhesive resin does not only remove the exposed collagen, produced by acid etching, but also solubilize the fibrils of the underlying mineralized matrix creating submicron porosities within the mineral phase. The adhesive resin will therefore infiltrate within the mineralized matrix filling these submicron porosities (i.e., creating a layer of resin-infiltrated mineralized matrix) [22]. Furthermore, the removal of exposed collagen does not only reduce the bonding technique sensitivity but also produce a more porous surface (i.e., an increase in size of dentinal tubules with the presence of an extensive labyrinth of lateral tubules that open on intertubular and/or peritubular dentin) [37] and larger resin tags [22] that would produce interfaces with high bond strength. The use of NaOCl also produced an acid-base resistant zone under the hybrid layer; this zone could help in resisting secondary caries around restorations [38].

### 3.2. Bond Strength Reduction

On the other hand, several mechanisms have been suggested for the adverse effect of NaOCl on dentin bond strength. It has been postulated that the reduction in bond strength after sodium hypochlorite treatment could be attributed to the removal of organic matrix from the treated dentin, leaving a less receptive bonding surface [39]. Furthermore, the removal of the organic matrix of dentin (i.e., dissolution of collagen fibrils from dentin by breakdown of the bonds between carbon atoms and disorganization of the primary structure of collagen) impedes the formation of a consistent hybrid layer [27]. After fragmentation of long collagen chains, it also chlorinates the protein terminal groups that are further broken down into smaller units [40]. The presence of protein chloramine-derived radicals in NaOCl-treated dentin could result in premature chain termination and hence incomplete polymerization of adhesive resins [41]. This means that sodium hypochlorite acts as an oxidizing agent and accordingly interferes with the free radical polymerization of the resin cement at the resin-dentin interface [42]. Another possible cause for the reduction in bond strength has been attributed to the presence of residual irrigants and/or their products that can easily diffuse into the dentin via dentinal tubules. These residual chemicals may contaminate the dentin surface and interfere with the penetration of resin adhesive into the dentin or the polymerization of resin monomer [27]. Even with complete penetration of resin adhesive into treated dentin, the bond strength is still reduced. This emphasizes the importance of the integrity of collagen fibrils left after acid etching and the quality of the hybrid layer (intermingling of adhesive resin with collagen fibrils) in dentin bonding [29]. Furthermore, the reduction in both calcium and phosphorus ions in dentin [43] and hence a reduction in dentin strength and elasticity [44, 45] could be responsible for the reduction in bond strength. The deterioration effect of sodium hypochlorite on structural, chemical, and mechanical properties of dentin is concentration-dependent. For example, 1% NaOCl had no significant adverse effect on carbon and nitrogen content as well as elasticity and flexural strength of dentin in contrast to 5 and 9% [45].

## 4. Reversing the Adverse Action of Sodium Hypochlorite on Dentin Bond Strength

The negative action of NaOCl on dentin bond strength can be reversed by some natural antioxidants, for example, ascorbic acid [30], sodium ascorbate [30, 39, 46], rosmarinic acid [46], green tea extracts [47], and proanthocyanidin [48]. These antioxidants could improve the bond strength to NaOCl-treated dentin and stabilize the resin-dentin interface due to their antioxidative capacities. They also remove the remnant of sodium hypochlorite by oxidation-reduction reaction. They have been therefore introduced as polymerization-facilitating agents and cross-linkers [46].

### 4.1. Ascorbic Acid and Sodium Ascorbate

L-ascorbic acid is water-soluble vitamin C; sodium ascorbate is a salt of ascorbic acid. Both are known for their antioxidant action. Stevens 2014 observed that a rinse with 10% sodium ascorbate (for 5 and 60 s) after NaOCl provided an immediate restoration of at least 50% of the original bond strength with the susceptible resin cements (self-etching, self-adhesive) [24]. The application of 10% sodium ascorbate for 10 min on NaOCl-treated dentin enhanced the bond strength of the total-etch system [28]. 10% sodium ascorbate also improved the degree of conversion and bond strength of RealSeal to NaOCl-treated dentin [49]. The reduction in bond strength of Metabond resin cements was also reversed by the application of either 10% sodium ascorbate (from 1–10 min) [30, 39, 46] or 10% ascorbic acid [30]. With the 4-META/MMA-TBB resin, 10 wt% ascorbic acid or 5 wt% sodium thiosulfate solution is used to reverse the action of sodium hypochlorite [31].

The action of sodium ascorbate was more effective in improving the bond strength than ascorbic acid [30]. Unlike ascorbic acid, sodium ascorbate has reducing action on root dentin and hence does not interfere with the polymerization of methyl methacrylate in resin cements used to fill the root canal [30]. It has been also suggested that sodium ascorbate can promote the polymerization reaction of adhesive resin without premature termination caused by NaOCl and reverses the disrupted bonding to NaOCl-treated dentin [50].

### 4.2. Rosmarinic Acid

Rosmarinic acid is a polyphenolic compound that can be extracted from the rosemary plant; it is a potent antioxidizing agent [51, 52] and matrix metalloproteinases (MMP, involved in the degradation process of hybrid layer) inhibitor [53]. The application of 100 *μ*M rosmarinic acid solution for 5 or 10 s improved the microtensile bond strengths to 6% NaOCl-treated dentin (30 s exposure); in this study, however, 10% sodium ascorbate solution was not effective in reversing the reduced pull-out bond strength [46]. In another study, 10% rosmarinic acid applied for 2 min was effective in reversing the reduced action of NaOCl on dentin and 10% sodium ascorbate hydrogel for 2 min was more effective than rosmarinic acid [34].

### 4.3. Green Tea Extract

Green tea extract is made from* Camellia sinensis*; it is composed of polyphenols, called catechins, as epicatechin, epicatechin gallate, epigallocatechin, and epigallocatechin gallate. Both epicatechin gallate and epigallocatechin gallate are characterized by their MMP inhibition action [54]. It also has antibacterial action; therefore, it has been proposed as root canal irrigant [55, 56]. Therefore, in addition to its antioxidant action, the application of 20 *μ*l of 2% green tea extract for 60 s was effective in increasing the bond durability of etch and rinse system to dentin [57].

### 4.4. Proanthocyanidin (Grape Seed Extract)

Proanthocyanidin has been found in high concentration in grape seed extract, pin bark extract, cocoa beans, lemon tree bark, and hazel nut tree leaves [48]. It has been observed that the antioxidant ability of proanthocyanidin is 20 times higher than that reported for sodium ascorbate; its absorbtion can be quicker and more complete than sodium hypochlorite [58]. The action of 5% proanthocyanidin is more effective than 10% sodium ascorbate in reversing the action of sodium hypochlorite [48]. Gallic acid, observed in proanthocyanidin, could play an important role in enhancing the bond strength. Finally, the ability of proanthocyanidin to cross-link and hence stabilize collagen could also increase the bond strength. All these factors could contribute to the strong reversal action of proanthocyanidin on the negative action of sodium hypochlorite [48].

It has been observed that proanthocyanidin enhanced the bond strength and durability of resin based sealers to root canal dentin after short storage in water; it also improved the resistance of demineralized dentin to degradation [59]. The inclusion of proanthocyanidin into dental adhesives, however, reduced the bond strength particularly when incorporated at high concentration (3%) [60]. Furthermore, the incorporation of proanthocyanidin into the adhesive produced a significant effect in reducing the bond strength compared to when it is incorporated into the primer [61].

## 5. Concluding Remarks

Under the light of this review, standardization of dentin source, preparation techniques, and testing methods is critical for assessing the action of NaOCl on dentin. Furthermore, simulating as much as possible the clinical scenario during which the material is used is another factor to be considered in conducting any relevant study. Clinically, NaOCl is used to irrigate the root canal system (i.e., confined environment), but* in most *in vitro studies, covered in this review, it is applied to flat dentin discs (i.e., unconfined environment). The penetration of NaOCl and hence its effect on dentin in both cases will be different. Additionally, NaOCl has been constantly applied for 10–20 min or even longer periods (hours in some studies) and this could not be achieved in clinical scenarios. Therefore, it would be expected that the action of NaOCl in these studies would be more aggressive than what happens in reality.

Regardless of the conflict seen in literature about the action of sodium hypochlorite on dentin bond strength, some natural antioxidants, for example, ascorbic acid, sodium ascorbate, rosmarinic acid, green tea extracts, and proanthocyanidin, could be very effective in modifying dentin surface and hence affecting its bond strength to other materials. In addition to their antioxidant action, some of these natural antioxidants have antibacterial action (e.g., green tea extracts) or work as matrix metalloproteinases inhibitors (e.g., rosmarinic acid). Therefore they could also increase the resistance of dentin to biodegradation and hence stabilize the resin adhesive-dentin interface by attacking any residual bacteria or inhibiting the degradation of the hybrid layer.

## Figures and Tables

**Table 1:** Summary of researches carried out to study the effect of sodium hypochlorite on bond strength to dentin.

Author	Technique	Form of NaOCl	Concentration of NaOCl (%)	Time of application of NaOCl (min)	Bond strength test	Source of dentin, location	Type of adhesive	Material bonded to dentin	Effect of NaOCl on bond strength in comparison to control
Wakabayashi et al [20]	After etching with 40% H2SO4	—	10	—	Tensile	—	—	—	Increase

Vargas et al. [21]	After etching with 37% H3PO4	—	5	2	Shear	Human 3rd molars, coronal dentin	All Bond 2 (AB2)	Z100 (composite resin)	Increase
							Scotchbond Multi-Purpose (SBMP)		No change

Prati et al. [22]	After etching with 35% H3PO4 for 20 s	Gel	1.5	2	Shear (immediate)	Human 3rd molars, coronal dentin	Optibond FL Scotchbond MP Single Bond Prime & Bond 2	Z100 (composite resin)	Increase Decrease Decrease No change

Ari et al. [23]	Before bonding	Solution	5	5	Microtensile	Human single rooted tooth, root canal dentin (after filling root canal, they were sectioned into 1 mm thick slabs from CEJ to apex)	C&B Metabond Panavia F Variolink II Rely-X	C&B Metabond Panavia F Variolink II Rely-X	Decrease No change

Stevens [24]	Before bonding	Solution	6	20	Shear (immediate)	Human molars & premolars, coronal dentin	Variolink II (total-etch)	Ceramic rod etched with 4.9% HFl acid for 20 s and silanated with Monobond Plus for 1 min	No change
							Clearfil SA Cement (self-etch)		Decrease
							Clearfil Esthetic Cement EX (self-etch)		No change
							Speed CEM (self-etch, self-adhesive)		Decrease
							Multilink (self-etch, self-adhesive)		No change

Ishizuka et al. [25]	Before bonding	Solution	6 v/v	1,5 & 10	Push-out shear	Bovine mandibular 1st & 2nd incisors, root canal dentin (after filling root canal with gutta-percha, a truncated cone cavity of 4 mm diameter & 2.5 mm height within coronal part of the root was prepared. Cavity was then filled and sectioned into 1 mm thick slabs)	Clearfil Mega Bond (self-etch primer) Single Bond (total-etch)	Clearfil AP-X (composite resin)	Decrease No change

Frankenberger et al. [26]	After etching	—	—	—	Push-out	Human 3rd molars, coronal dentin	(i) Scotchbond Multi-Purpose Plus (total-etch) (ii) EBS (tot al-etch) (iii) Solid Bond (total-etch) (iv) Prime & Bond 2.1 (self-etching, self- adhesive) (v) Syntac Sprint (self-etch, self -adhesive)	Each adhesive used with its corresponding composite resin	Decrease

Nikaido et al. [27]	Before bonding	—	5	1	Tensile	Bovine root canal dentin	Single Bond (total etch)	—	Decrease
							Superbond C&B (self-cure)		Decrease
							Clearfil Liner Bond II (self-etch primer)		No change

Vongphan et al. [28]	Before bonding	Solution	5.25	10	Microtensile	Human 3rd molars, pulp chamber dentin (cavity in pulp chamber was filled & sectioned vertically into 0.8 mm thick slabs that were trimmed into dumbbell shaped specimens)	Single Bond (total-etch)	Z250 (composite resin)	Decrease

Perdigão et al. [29]	After etching with 34% H3PO4 for 15s	Gel	10	1/4, 1/2, & 1	Shear	Bovine, coronal dentin	Prime & Bond NT (total-etch)	Surefil (composite resin)	Decrease with time
							Single Bond (total-etch)	Z100 (composite resin)	

Morris et al. [30]	Before bonding	Solution	5	15–20	Tensile	Human maxillary incisors and mixed canines, root canal dentin (after filling root canal, they were sectioned into 1 mm thick slabs from CEJ to apex)	C&B Metabond	C&B Metabond	Decrease

Soeno et al. [31]	After etching with 40% H3PO4	Gel	10	—	Tensile	Bovine, coronal dentin	Carboxylic (4-META/MMA-TBB) resin Superbond C&B (10-3/SB)	Sandblasted stainless steel rods	Decrease

CEJ: cementoenamel junction; HFl: hydrofluoric acid.
